# Evaluation of risk factors associated with the development of MDR- and XDR-TB in a tertiary care hospital: a retrospective cohort study

**DOI:** 10.7717/peerj.10826

**Published:** 2021-03-18

**Authors:** Amna Saifullah, Tauqeer Hussain Mallhi, Yusra Habib Khan, Muhammad Shahid Iqbal, Nasser Hadal Alotaibi, Abdulaziz Ibrahim Alzarea, Maria Rasheed

**Affiliations:** 1 Institute of Pharmacy, Lahore College for Women University, Lahore, Punjab, Pakistan; 2 Current Affiliation: Department of Pharmacy, University of Lahore, Chenab Campus, Gujrat, Pakistan; 3 Department of Clinical Pharmacy, College of Pharmacy, Jouf University, Sakaka, Al-Jouf, Saudi Arabia; 4 Department of Clinical Pharmacy, College of Pharmacy, Prince Sattam bin Abdulaziz University, Al-kharj, Saudi Arabia

**Keywords:** Tuberculosis, Drug resistance tuberculosis, Multi drug resistant tuberculosis, Extensively drug resistant tuberculosis, Risk factors, DOT

## Abstract

**Background:**

Drug resistant tuberculosis (DR-TB) infringes substantial burden in terms of longer treatment duration, morbidity and mortality. Timely identification of patients at risks of DR-TB will aid individualized treatment. Current study was aimed to ascertain several factors associated with DR-TB among patients attending a tertiary care hospital.

**Methods:**

This retrospective study was conducted among patients with confirmed diagnosis of DR-TB and drug susceptible TB (DS-TB) seeking medical care from a tertiary care hospital during 2014–2019. The types of DR-TB included were rifampicin resistant tuberculosis (RR-TB), Multidrug resistant tuberculosis (MDR-TB) and extensively drug resistant tuberculosis (XDR-TB). Appropriate statistical methods were implied to evaluate the factors associated with DR-TB.

**Results:**

Out of 580 patients, DS-TB was diagnosed in 198 (34.1%) patients while DR-TB was present in 382 patients. Of resistance cases, RR-TB, MDR-TB and XDR-TB were diagnosed in 176 (30.3%), 195 (33.6%) and 11 (1.9%) patients, respectively. Significant differences (*P* < 0.05) in demographics and clinico-laboratory characteristics were observed between patients with DS-TB and DR-TB. Logistic regression analysis revealed age ≤38 years (OR: 2.5), single marital status (OR: 11.1), tobacco use (OR: 2.9), previous treatment (OR: 19.2), treatment failure (OR: 9.2) and cavity on chest X-ray (OR: 30.1) as independent risk factors for MDR-TB. However, XDR-TB was independently associated with age group of ≤38 years (OR: 13.6), students (OR: 13.0), previous treatment (OR: 12.5), cavity on chest X-ray (OR: 59.6). The independent risk factors associated with RR-TB are age ≤38 years (OR: 2.8), females (OR: 5.7), unemployed (OR: 41.5), treatment failure (OR: 4.9), previous treatment (OR: 38.2) and cavity on chest X-ray (OR: 4.3). ROC curve analysis accentuate the excellent predictive accuracy of all logistic regression models as shown by AUC (0.968, *P* < 0.001) for MDR-TB, AUC (0.941, *P* < 0.001) for XDR-TB and AUC (0.962, *P* < 0.001) for RR-TB.

**Conclusions:**

Current study demonstrates a sizeable extent of resistant cases among pulmonary TB patients. This study presaged significant risk of DR-TB among females, young adults, unemployed, smokers, patients with previous treatment failure and cavitation on chest X-ray. Timely identification of high risk patients will give pronounced advantages regarding appropriate choices of prevention, treatment and disease control.

## Introduction

Despite global efforts to curb the growing burden of tuberculosis (TB), it is considered number one cause of deaths among infectious diseases, ranking above human immunodeficiency virus/acquired immunodeficiency syndrome (HIV/AIDS). According to an estimate, 10.4 million new cases of drug susceptible TB (DS-TB) and 1.67 million deaths due to TB were reported in 2016 around the globe (Floyd et al., 2018). Multidrug resistant TB (MDR-TB) and extensively drug resistant TB (XDR-TB) are major health concerns worldwide due to swift rise in resistant cases with corresponding increase in prevalence of TB. MDR-TB is a disease caused by resistant strains of *Mycobacterium tuberculosis (M. tuberculosis)* to two important first line anti-tuberculosis drugs (rifampicin and isoniazid) with or without concomitant resistance to other anti-tubercular drugs (Wang et al., 2014). XDR-TB, a subset of MDR-TB, is a disease associated with resistance to at least isoniazid, rifampicin, a fluoroquinolone, and any one of three injectable second-line drugs (amikacin, kanamycin, or capreomycin) (He et al., 2017). According to the World Health Organization (WHO) global TB report, the estimated proportion of MDR/Rifampicin resistant-TB (RR-TB) cases has reached to 3.4% of new cases and 18% of previously treated cases in 2018 (Floyd et al., 2018). Of these MDR-/RR-TB cases, 109,699 cases had XDR-TB. However, this proportion is higher than global average in some countries (Floyd et al., 2018; World Health Organization, 2017). Pakistan ranks 5th amongst eighteen highest TB burden countries, contributing around 61% of cases in the WHO Eastern Mediterranean Region (EMR) (World Health Organization, 2019). According to TB country profile 2019 produced by WHO EMR office (EMRO), the incidence of TB disease in Pakistan is more than 265 per 100,000 population. This profile estimates that total notified cases in Pakistan are 369,458 accounting for 20% of extra pulmonary and 80% of pulmonary TB (PTB). The incidence rate of MDR-/RR-TB is 13 cases per 100,000 population. (4.2% new cases and 16% previously treated cases). Pakistan is also ranked fourth among highest incidence of MDR-TB countries (World Health Organization, 2019). During the years 2006–2009, the prevalence of XDR-TB in Pakistan has increased from 1.5% to 4.5% (Hasan et al., 2010). The management and treatment of MDR- and XDR-TB is complex enough that it becomes difficult to achieve desirable treatment outcomes as compared to DS-TB (Atif et al., 2017). Several factors contribute to the high prevalence of drug resistance TB (DR-TB) including lack of effective TB control programme, illiteracy, poverty (Ullah et al., 2016) and limited research elaborating the factors associated with DR-TB. Few studies were conducted to explore the factors of MDR-TB in Pakistan. Ahmad et al. (2012) investigated age, gender, educational status, Sindhi ethnicity, TB contacts and prior TB treatment as significant risk factors of MDR-TB in urban population of Karachi. However, the findings of their study are limited in generalizability to rural population and to other provinces of Pakistan. Punjab is a most populous, prosperous, and developed province of Pakistan with an estimated 60% of the country’s population. It requires thorough investigation and evaluation of risk factors contributing towards DR-TB in the province. Few studies conducted in Punjab have not strived to couple clinical, microbiological and radiological findings with MDR-TB compared to DS-TB (Ullah et al., 2016; Tabassum et al., 2018). In addition, to the best of our knowledge, there is no study conducted in Pakistan to determine risk factors of XDR-TB. The identification and assessment of factors associated with RR-, MDR- and XDR-TB are crucial for commencement of targeted drug susceptibility testing (DST), early initiation of second line drugs for high risk patients and development of cost effective strategies to control DR-TB in Pakistan (Wang et al., 2014). These risk factors could also be used in formulating predictive score to identify high risk DR-TB patients and to prioritise care that may aid in reduced morbidity and mortality. In these contexts, a retrospective case control study was conducted to identify the factors associated with MDR-, XDR- and RR-TB among patients attending a tertiary care hospital in Pakistan.

## Subjects and methods

### Ethics statement

The study was approved by the Office of the Medical Superintendent, Allama Iqbal Memorial Hospital (registration number: AIMTH/IEB/2018/564). The need for patient consent was waived by the Office of the Medical Superintendent, Allama Iqbal Memorial Hospital Sialkot, due to the retrospective nature of the study. All the relevant information of study participants was routinely collected, recorded and managed by attending physicians, directly observed treatment short course (DOTS) facilitator and PMDT data manager.

### Study setting

Sialkot is one of the districts of the Punjab province of Pakistan. It is located in the north east of the province with an area of 3,016 km^2^ with an estimated population of 3.894 million. Current study was conducted at Pulmonology Department of Allama Iqbal Memorial Teaching Hospital (AIMTH) in Sialkot. AIMTH is a 400 beds teaching hospital. The patients treated in this hospital are either self-referral or referred by the physicians from other public or private hospitals and community clinics of the city or other nearby districts.

### Study population

All the patients with confirm diagnosis of DS- and DR- PTB attending pulmonology department of AIMTH during October 2014–January 2019 were included in the current study. This duration of patient’s recruitment was selected because Programmatic Management of drug resistant tuberculosis (PMDT), a step towards support of new STOP TB strategy, was established in 2014 in the hospital.

### Inclusion/exclusion criteria

**Figure 1:**
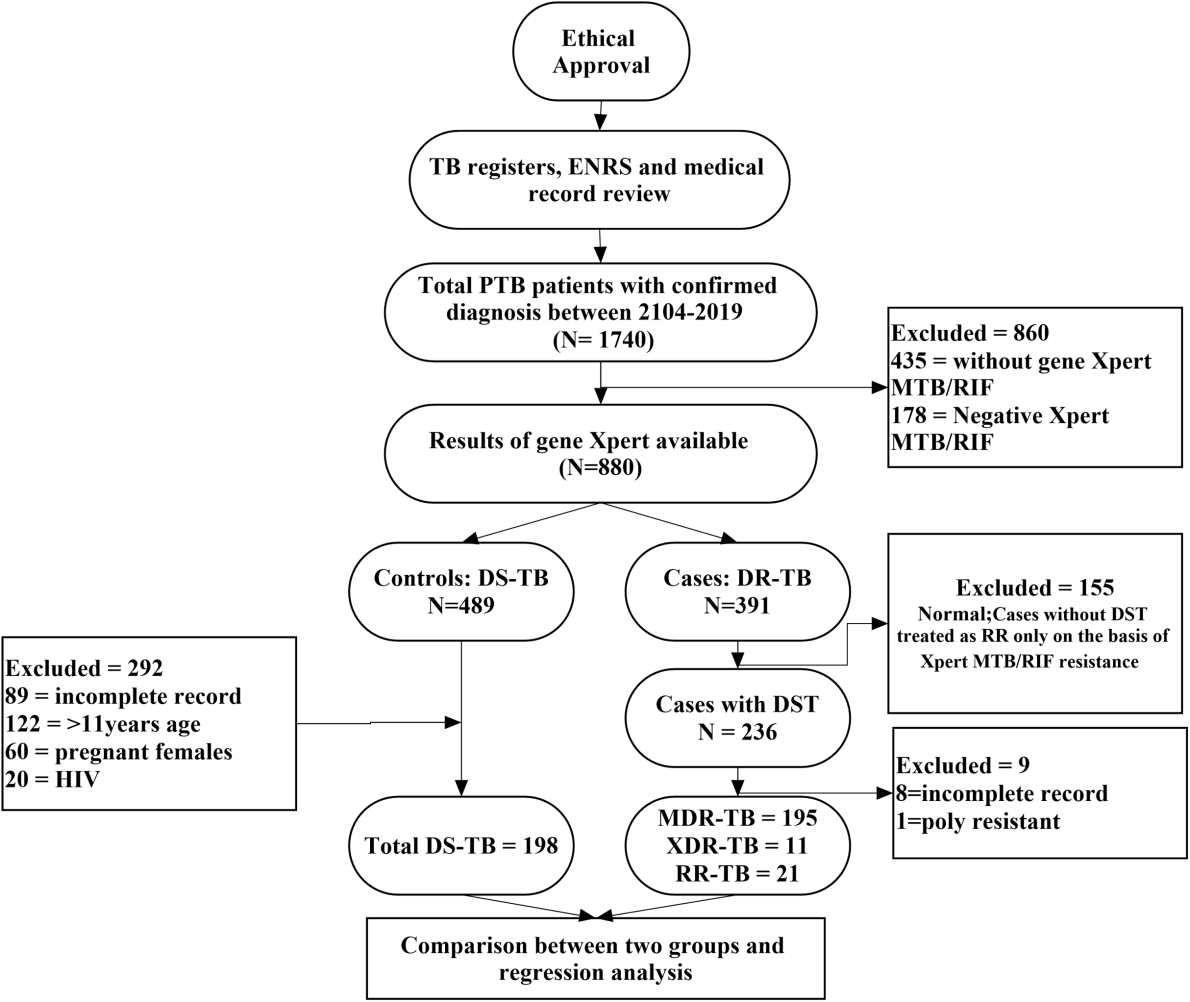
Methodological flow chart of the study.

In present study, a case was considered any PTB patient of either sex, aged 11 years or above and diagnosed with Xpert MTB/RIF proven RR, DST culture proven RR-, MDR- or XDR-TB. A control was a PTB patient of either sex, aged 11 years or above and DST culture confirmed DS-TB patient (Ullah et al., 2016; Ahmad et al., 2012; Balaji et al., 2010). Sputum smear negative DS-TB patients, HIV patients, pregnant females or patients with incomplete medical and microbiological records were excluded from the analysis (Desalegn et al., 2018). However, sputum smear negative but culture positive resistant MDR- and XDR-TB patients were included in the final analysis because acid fast bacilli (AFB) sputum smear often shows negative results due to low count of bacteria and positive culture confirms the diagnosis (Linguissi et al., 2015). HIV patients were not included into the study due to referral of substantial number of patients to the other speciality care hospitals. Moreover, pregnant females were deliberately excluded to avoid any risk of bias related to physiological changes during pregnancy. The methodological flow diagram of the current study is described in Fig. 1.

### Data collection and management

All the required data of patients were extracted from Medical and Electronic Nominal Recording Reporting System (ENRS). A pre-designed data collection form (DCF) was used to extract all the relevant information. The primary components of DCF were demographics, including gender, age, initial weight, occupation, marital status and residential status. Social history included tobacco use, alcohol and drug abuse. Clinical characteristics were comprised of previous treatment history of TB, outcomes of previous treatment, family history of TB, presence of cavity on chest X-ray (CXR), sputum smear positivity at onset and any co-morbid conditions including diabetes, hypertension, chronic obstructive pulmonary disease (COPD), gastric ulcer and hepatitis C.

### Bacteriology and DST

In compliance with the guidelines of national TB control program (NTP), all “presumptive TB cases” with suspected CXR at TB outpatient department were subjected to direct sputum smear microscopy and rapid DST including Xpert MTB/RIF. Sputum smear microscopy was performed with two sputum samples (at the spot sputum sample and next morning sample) to identify the presence of AFB by using the Ziehl-Neelsen stain. Xpert MTB/RIF was performed to detect *M. tuberculosis* complex and RR simultaneously. Patients who were declared rifampicin sensitive were enrolled for further DS anti-TB treatment. The patients who were RR detected, referred to PMDT site for the treatment. Their specimen was sent to TB reference laboratory, where the culture test was carried out on Lowenstein-Jensen medium. All positive cultures were subjected to DST using the agar proportion method performed on enriched middle-brook 7H10 medium against all first line (except pyrazinamide) and second line anti-TB drugs. BACTEC mycobacterial growth indicator tube (MGIT) was used for DST of pyrazinamide.

### Case definitions

DS-TB case was defined as TB patient who had DST confirmed susceptibility to anti-TB drugs (Ahmad et al., 2012). RR-TB: A Disease detected as resistance to rifampicin with or without resistance to other anti-TB drugs. MDR-TB; A disease caused by resistant strains of *M. tuberculosis* to two important first line anti-TB drugs rifampicin and isoniazid with or without concomitant resistance to other anti-TB drugs (Wang et al., 2014). XDR-TB; A disease associated with resistance to at least isoniazid, rifampicin, a fluoroquinolone and any one of three injectable second-line drugs (amikacin, kanamycin, or capreomycin) (He et al., 2017). Registration group of patients at PMDT site were based upon previous treatment outcomes. These treatment outcomes were defined with minor modification to the definitions given by WHO and International Union against TB and Lung Disease (IUATLD) guidelines (World Health Organization, 2011; Laserson et al., 2005). These definitions were; New TB case: An incident tubercular infection case that met the suspected or confirmed case criteria and who had denied any previous TB diagnosis or anti-TB drugs treatment for 30 days or less (Tang et al., 2013); previously treated TB case: a patient who had received anti-TB drug treatment for at least one month in past and who had documented evidence of previous treatment in medical and electronic records (Laserson et al., 2005; Liu et al., 2011); relapse: a patient who had previous anti-TB treatment declared cured or completed but reported again with a positive sputum smear; treatment after failure: a patient who had a positive sputum smear at the end of 5th month of treatment for category I patient and who had 2 or more positive cultures of total five cultures in the final 12 months of treatment or any one positive cultures of the final three cultures for DR-TB patient; defaulter: a patient in which previous treatment was interrupted by 2 or more consecutive months. Others previously treated registration group: A patient who had previously been treated against TB but outcome of his previous treatment was unknown or not documented.

### Statistical analysis

All data were analysed using SPSS 22.0 for windows (SPSS Inc., Australia). Quantitative variables were intimated as mean ± SD, while qualitative data were denoted as number of observations with percentages. Comparisons of categorical variables were performed by using χ^2^ test or Fisher’s exact test, where appropriate. To evaluate the strength of association between 2/2 and greater than 2/2 variables, Phi coefficient and Cramer’s V were used. The association was considered strong, moderate or weak, if Phi coefficient and Cramer’s V values were (0.5–1), (0.3–0.49) and (0.1–0.29), respectively (Mallhi et al., 2015). For the comparison of continuous variable, student-*t* test was performed. A logistic regression model was used to determine risk factors independently associated with MDR- and XDR-TB. Variables with *p* value < 0.05 in univariate analysis were subjected to multivariate logistic regression. Adjusted OR and 95% CI were calculated. Receiver operating curve (ROC) analyses was performed to observe how the predicted model can distinguish between true positives and negatives. To assess the calibration of final multivariable logistic regression model, hosmer-lameshow test was probed. A double sided *p*-value < 0.05 was used to consider variables as statistically significant.

## Results

A total of 1,740 PTB cases were identified retrospectively which subsequently subjected to exclusion of 1,160 patients; 860 patients did not have Xpert MTB/RIF performed or negative results, 291 were TB patients with age <11 years, HIV positive, pregnant females or unavailability of medical records and nine patients were DST confirmed resistant patients with poly resistance or incomplete medical records. A total of 580 patients were included in the final analysis (Fig. 1).

### Socio demographic characteristics of DS-, MDR-and XDR-and RR-TB patients

According to DST criteria, DS-TB was diagnosed in 198 (34.1%) patients while MDR- and XDR-, RR-TB were diagnosed in 195 (33.6%), 11 (1.9%) and 176 (30.3%) patients, respectively. Out of total 198 DS-TB controls, 42.4% were females with a mean age of 40.94 ± 19.05 years, whereas majority (*n* = 106 (54.4%)) of MDR-TB cases were male with a mean age of 35.87 ± 15.93 years. With the exception of gender, statistically significant differences were observed among the demographic profile of both groups (Table 1). About 63% cases of XDR-TB were males, with a mean age of 27.18 ± 10.08 years. The characteristics of XDR-TB cases and DS-TB controls were compared and significant differences were observed in age and occupation with XDR-TB cases being younger than DS-TB controls 241 (Table 1).

**Table 1:** Socio-demographic characteristics of DS-, MDR- and XDR-TB patients.

Characteristics	Total *n* = 580	DS-TB *n* = 198	MDR-TB *n* = 195	*P* values	XDR-TB *n* = 11	*P* values	RR-TB *n* = 176	*P* values
Gender				0.521^1^^,^**		0.764^1^		**0.034**^2^^,^**
Male	309 (53.3%)	114 (57.6%)	106 (54.4%)		7 (63.6% )		82 (46.6%)	
Female	271 (46.7%)	84 (42.4%)	89 (45.6%)		4 (36.4% )		94 (53.4%)	
Age***	37.63 ± 17.6	40.94 ± 19.05	35.87 ± 15.926	**0.001**	27.18 ± 10.08	**0**.**001**	36.52 ± 17.4	**0.019**
Weight***	47.49 ± 11.4	49.45 ± 11.01	45.59 ± 11.15	**0.004**	45.82 ± 10.41	0.286	47.47 ± 11.8	1.983
Marital status								
Single	235 (40.5%)	58 (29.3%)	132 (67.7%)	**<0.001**^3^	6 (54.5%)	0.096^2^	59 (33.5%)	0.379^1^
Married	314 (54.1%)	120 (60.6%)	63 (32.3%)	**<0.001**^2^^,^**	5 (45.5%)	0.355^1^^,^**	106 (60.2%)	0.940^1^^,^**
Widowed	31 (5.3%)	20 (10.1%)	–		–		11 (6.3%)	0.178^1^^,^**
Occupation								
Unemployed	96 (16.6%)	38 (19.2%)	14 (7.2%)	**<0.001**^2^^,^**	–		44 (25%)	**<0.001**^4^
Student	101 (17.4%)	27 (13.6%)	37 (19%)	0.152^2^	5 (45.5%)	**0.015**^2^	32 (18.2%)	0.229^1^
Housewife	167 (28.8%)	52 (26.3%)	55 (28.2%)	0.665^1^	2 (18.2%)	0.732^1^^,^**	58 (33%)	0.156^1^
Businessman	9 (1.6%)	9 (4.5%)	–		–		–	
Labour	186 (32.1%)	58 (29.3%)	86 (44.1%)	**0.002**^2^	2 (18.2%)	0.733^1^^,^**	40 (22.7%)	0.150^1^^,^**
Others	21 (3.6%)	14 (7.1%)	3 (1.5%)	**0.007**^2^^,^**	2 (18.2%)	0.201^2^	2 (1.1%)	**0.005**^2^^,^**
Residential area				**0.003**^2^^,^**		0.102^2^^,^**		**<0.001**^2^^,^**
Rural	429 (74%)	124 (62.6%)	149 (76.4%)		10 (90.9%)		146 (83%)	
Urban	151 (26%)	74 (37.4%)	46 (23.6%)		1 (9.1% )		30 (70%)	
Tobacco use	225 (38.8%)	86 (43.4%)	113 (57.9%)	0.**004**^2^	3 (27.3%)	0.360^2^^,^**	59 (33.5%)	**0.050**^2^^,^**
Alcohol use	10 (1.7%)	–	8 (4.1%)		–		2 (1.1%)	–
Drug abuse	8 (1.4%)	–	6 (3.1%)		–		2 (1.1%)	–

**Notes:**

** Negative Phi coefficient value.

*** Student-*t* test.

1 Phi coefficient (0.0).

2 Phi coefficient (0.1–0.29).

3 Phi coefficient (0.3–0.49).

4 Phi coefficient (0.5–1.0).

Values of categorical variables are shown as percentages (%) whereas continuous variables are presented as mean ± SD.

*P* values of categorical variables are calculated by χ^2^ test or fisher’s exact test, where appropriate.

*P* values for continuous variables are calculated by student-*t* test.

*P* values were calculated between patients with and without resistant disease.

The bold texts in the table represents statistically significant values.

In contrast to MDR- and XDR-TB, majority of RR-TB cases were females (53.4%) with a mean age of 36.52 ± 17.4 years. A statistically significant relationship was observed in gender, mean age, unemployment, other occupations, residential area and tobacco use among RR-TB cases.

### Comparison of clinical features between DS-, MDR- XDR- and RR-TB

Among the resistant cases, 13.3% of MDR- and 27.3% of XDR-TB cases were smear negative. 1 +ve sputum smears were more profound in DS-TB, 2+ smears were most detected in RR-TB TB and 3+ sputum smears were significantly associated with XDR-TB. DR-TB patients were most observed with cavitation on CXR accounting for 35.2% of RR-, 87.2% of MDR- and 90.9% of XDR-TB cases. Almost an equal proportion among DS- (21.2%) and MDR-TB (23.1%) patients was observed with family history of TB while RR- and XDR-TB had shown slightly greater percentage (33.5% and 36.4% respectively). Greater number of the DR-TB patients belonged to previously treated group. Out of DR-TB patients with previous treatment history, majority were registered as others previously treated group followed by relapse and lost to follow up. However, failure group was not prevalent among XDR-TB patients. Diabetes, hypertension and gastric ulcer were found to be equally distributed among DS-TB controls and RR-TB cases while other comorbidities were more profound among DS-TB patients. We also observed no comorbidities among patients with XDR-TB (Table 2).

**Table 2:** Comparison of clinical characteristics among patients with DS-, MDR- and XDR-TB.

Characteristics	Total *n* = 580	DS-TB *n* = 198	MDR-TB *n* = 195	*P*	XDR-TB *n* = 11	*P*	RR-TB *n* = 176	*P*
Smear positivity at the start of treatment								
Negative	29 (5%)	–	26 (13.3%)	–	3 (27.3%)	–	–	
1+ve	335 (57.8%)	126 (63.6%)	100 (51.3%)	**0.013**^2^^,^**	2 (18.2%)	**0.004**^2^^,^**	107 (60.8%)	0.572^1^^,^**
2+ve	173 (29.8%)	56 (28.2%)	53 (27.2%)	0.807^1^^,^**	3 (27.3%)	1.00^1^^,^**	61 (34.7%)	0.184^1^
3+ve	43 (7.4%)	16 (8.2%)	16 (8.2%)	0.964^1^^,^**	3 (27.3%)	**0.031**^1^	8 (4.5%)	0.164^1^^,^**
Radiological findings at onset				**<0.001**^4^		**<0.001**^4^		
Cavitary	269 (46.4%)	27 (13.6%)	170(87.2%)	–	10 (90.9%)	**-**	62 (35.2%)	**<0.001**^**2**^
Non-cavitary	311 (53.6%)	171 (86.4%)	25 (12.8%)	–	1 (9.1%)	–	114 (64.8%)	
Family history of TB	150 (25.9%)	42 (21.2%)	45 (23.1%)	0.656^1^	4 (36.4%)	0.263^2^	59 (33.5%)	**0.007**^2^
Registration group				**<0.001**^4^		**<0.001**^4^		
New	184 (31.7%)	149 (75.3%)	18 (9.2%)	**<0.001**^4^^,^**	1 (9.1%)	**<0.001**^3^^,^**	16 (9.1%)	**<0.001**^4^^,^**
Relapse	98 (16.9%)	40 (20.2%)	36 (18.5%)	0.662^1^^,^**	3 (27.3%)	0.700^1^	19 (10.8%)	**0.013**^2^^,^**
Lost to follow-up	43 (7.4%)	6 (3.0%)	19 (9.7%)	**0**.**006**^2^	1 (9.1%)	0.319^2^	17 (9.7%)	**0.008**^2^
Treatment failure	81 (14%)	3 (1.5%)	47 (24.1%)	**<0.001**^3^	–	–	31 (17.6%)	**<0.001**^2^
Others previously treated	174 (30.0%)	–	75 (38.5%)		6 (54.5% )		93 (52.8%)	–
Previous treatment				**<0.001**^4^		**<0.001**^3^		
Yes	396 (68.3%)	49 (24.7%)	177 (90.8%)		10 (90.9% )		160 (90.9%)	**<0.001**^4^
No	184 (31.7%)	149 (75.3%)	18 (9.2%)	–	1 (9.1%)	–	16 (9.1%)	
Comorbidities								
Diabetes	137 (23.6%)	54 (27.3%)	39 (20%)	**0.043**^2^^,^**	–	–	44 (25%)	0.410^1^^,^**
Hypertension	93 (16%)	43 (21.7%)	12 (6.2%)	**<0.001**^2^^,^**	–	–	38 (21.6%)	0.976^1^^,^**
COPD	16 (2.8%)	12 (6.1%)	2 (1%)	**0.007**^2^^,^**	–	–	2 (1.1%)	**0.012**^2^^,^**
Hepatitis C	25 (4.3%)	13 (6.6%)	11 (5.6%)	0.702^2^^,^**	–	–	1 (0.6%)	**0.002**^2^^,^**
Gastric ulcer	13 (2.2%)	4 (2%)	4 (2.1%)	0.983^1^^,^**	–	–	5 (2.8%)	0.605^1^

**Notes:**

** Negative Phi coefficient value.

1 Phi coefficient (0.0).

2 Phi coefficient (0.1–0.29).

3 Phi coefficient (0.3–0.49).

4 Phi coefficient (0.5–1).

Values of categorical variables are shown as percentages (%), while *p* values of categorical variables are calculated by χ^2^ test or fisher’s exact test, where appropriate.

The bold texts in the table represents statistically significant values.

Upon statistical comparison between DS-TB controls and RR-TB cases, significant differences were observed in radiological findings at onset (*p* < 0.001), family history of TB (*p* = 0.007), registration group (*p* < 0.001), previous treatment history (*p* < 0.001), COPD (*p* = 0.012) and hepatitis C (*p* = 0.002). Moreover, we found significant differences in 1+ sputum smear (*p* = 0.013), radiological findings at onset (*p* < 0.001), registration group (*p* < 0.001), previous treatment history (*p* < 0.001), diabetes (*p* = 0.043), hypertension (*p* < 0.001) and COPD (*P* = 0.007) when compared between DS- controls and MDR-TB cases. Current analysis found that the development of XDR TB among PTB patients was significantly associated with 1+ (*p* = 0.004) and 3+ (*p* = 0.031) sputum smears, radiological findings at onset (*p* < 0.001), registration group (*p* < 0.001), and previous treatment history (*p* < 0.001) as compared to those patients who did not develop DR-TB (Table 2).

### Pattern of resistance to anti tuberculosis drugs among MDR- and XDR-TB patients

We assessed pattern of resistance to both first line and second line anti-TB drugs among MDR-and XDR-TB patients. Almost an equal proportion of resistance to ethambutol was observed among both groups while no MDR-TB patient had shown resistance to amikacin. A significant difference was observed in resistance to second line anti-TB drugs (amino glycosides and flouroquinolones) (Table 3).

**Table 3:** Univariate and multivariate analysis to evaluate risk factors of MDR-TB.

Drugs	MDR-TB *n* = 195	XDR-TB *n* = 11	*P* value
Ethambutol	80 (41%)	5 (45.5%)	0.76^1^
Pyrazinamide	45 (23.1%)	5 (45.5%)	0.14^2^
Streptomycin	67 (34.4%)	5 (45.5%)	0.52^1^
Amikacin	0	8 (72.7%)	**<0.001^4^**
Kanamycin	1 (0.5%)	10 (90.9%)	**<0.001^4^**
Ofloxacin	66 (33.8%)	11 (100%)	**<0.001^3^**
Moxifloxacin	7 (3.6%)	4 (36.4%)	**0.007^2^**
Levofloxacin	22 (11.3%)	5 (45.5%)	**0.001^3^**

**Notes:**

1 Phi coefficient (0.0).

2 Phi coefficient (0.1–0.29).

3 Phi coefficient (0.3–0.49).

4 Phi coefficient (0.5–1.0).

Values of categorical variables are shown as percentages (%).

*P* values of categorical variables are calculated by χ2 test or fisher’s exact test, where appropriate.

*P* values were calculated between patients with MDR- and XDR-TB.

The bold texts in the table represents statistically significant values.

### Risk factors associated with MDR-TB

**Figure 2:**
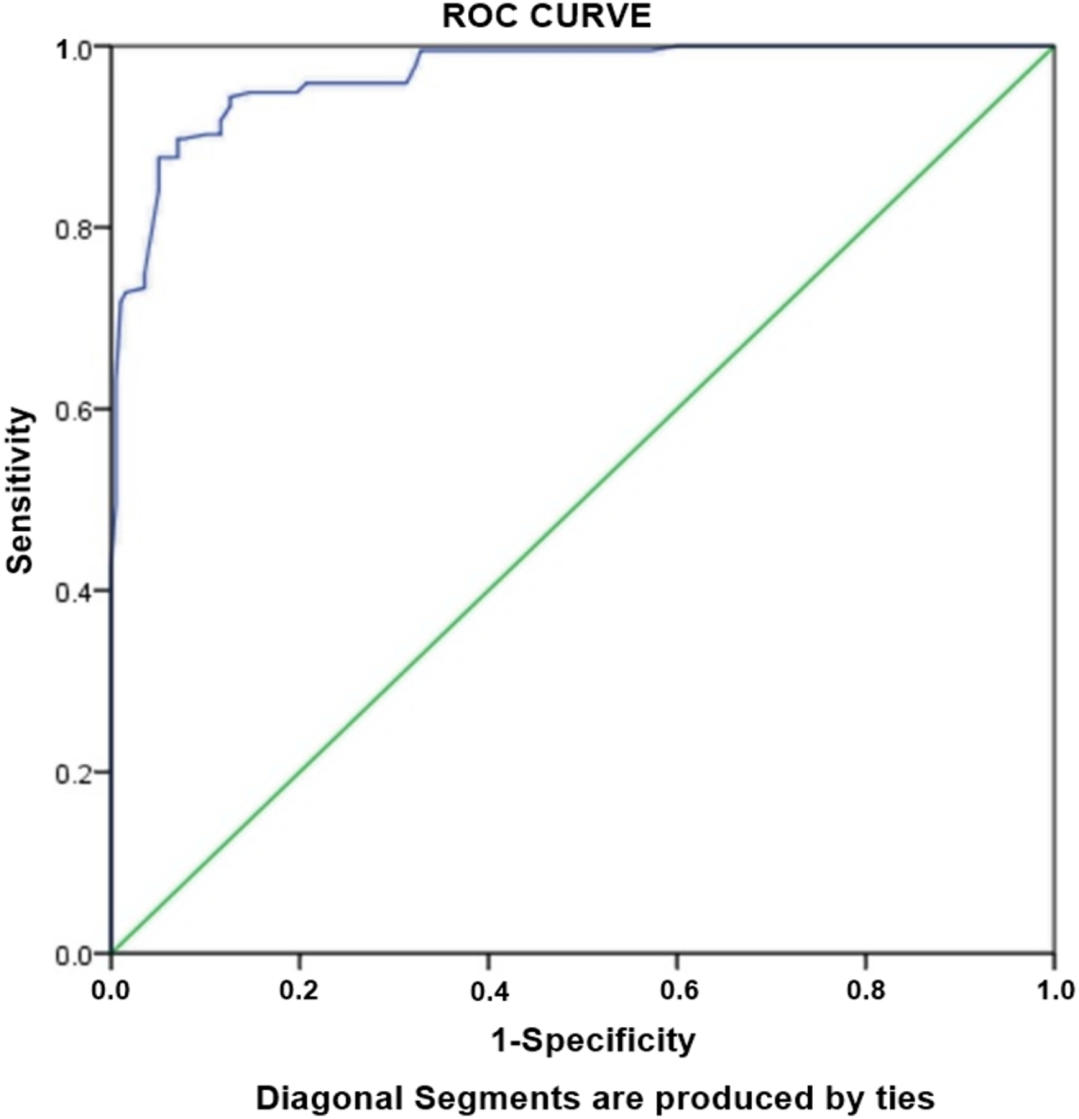
Receiver-operating characteristics curve analysis of multivariate logistic model predicting risk factors of MDR-TB. AUC, area under the curve AUC: 0.968 (95% CI [0.953–0.982], *P* < 0.001).

Logistic regression analysis was performed to assess the association between various factors with MDR-TB. Unadjusted univariate analysis revealed ten independent predictors of MDR-TB which were further subjected to multivariate analysis (Table 4). The adjusted analysis was created using the variables with *p* < 0.05. Multivariate regression model demonstrated cavity on CXR as strongest factor (OR: 30.1) of MDR-TB, followed by previous treatment (OR: 19.2) and single marital status (OR: 11.1). Other factors significantly associated with the MDR-TB were age ≤38 years (OR: 2.5), tobacco use (OR: 2.9) and failure to the previous treatment (OR: 9.2) (Table 4). Hosmer-Lameshow test statistics have shown χ2: 11.250, degree of freedom: 8 and *p* value = 0.188. The *p* value > 0.05 demonstrated that model is good fit to data (there is no difference between observed and predicted (model) values of the dependent variables). ROC curve analysis with AUC as 0.968 (95% CI [0.953–0.982], *P* < 0.001) demonstrated excellent predictive ability for regression model of MDR-TB (Fig. 2).

**Table 4:** Univariate and multivariate analysis to evaluate risk factors of XDR-TB.

Variables	Univariate			Multivariate		
	*P* value	uOR	95% CI	*P*	aOR	95% CI
Age						
≤38 years	0.038	1.5	[1.0–2.3]	0.033	2.5	[1.0–6.2]
Weight						
≤50kg	0.013	1.7	[1.1–2.6]	–	–	–
Marital status						
Single	<0.001	5.0	[3.3–7.8]	<0.001	11.1	[4.6–26.6]
Area						
Rural	0.003	1.9	[1.2–2.9]	–	–	–
Occupation						
Labour	0.002	1.9	[1.2–2.8]	–	–	–
Tobacco use	0.004	1.7	[1.2–2.6]	0.016	2.9	[1.2–6.7]
Registration group						
Lost to follow-up	0.010	3.4	[1.3–8.8]	–	–	–
Failure	<0.001	20.6	[6.3–67.6]	0.005	9.2	[1.9–42.4]
Previous treatment	<0.001	29.9	[16.7–53.5]	<0.001	19.2	[8.2–46.1]
Cavity on CXR	<0.001	43.0	[24.0–77.2]	<0.001	30.1	[13.4–67.7]

**Note:**

Hosmer-Lameshow test statistics: χ2: 11.250, degree of freedom: 8, *p* value = 0.188. Only significant associations shown, CXR: chest x-ray

### Risk factors associated with XDR-TB

**Figure 3:**
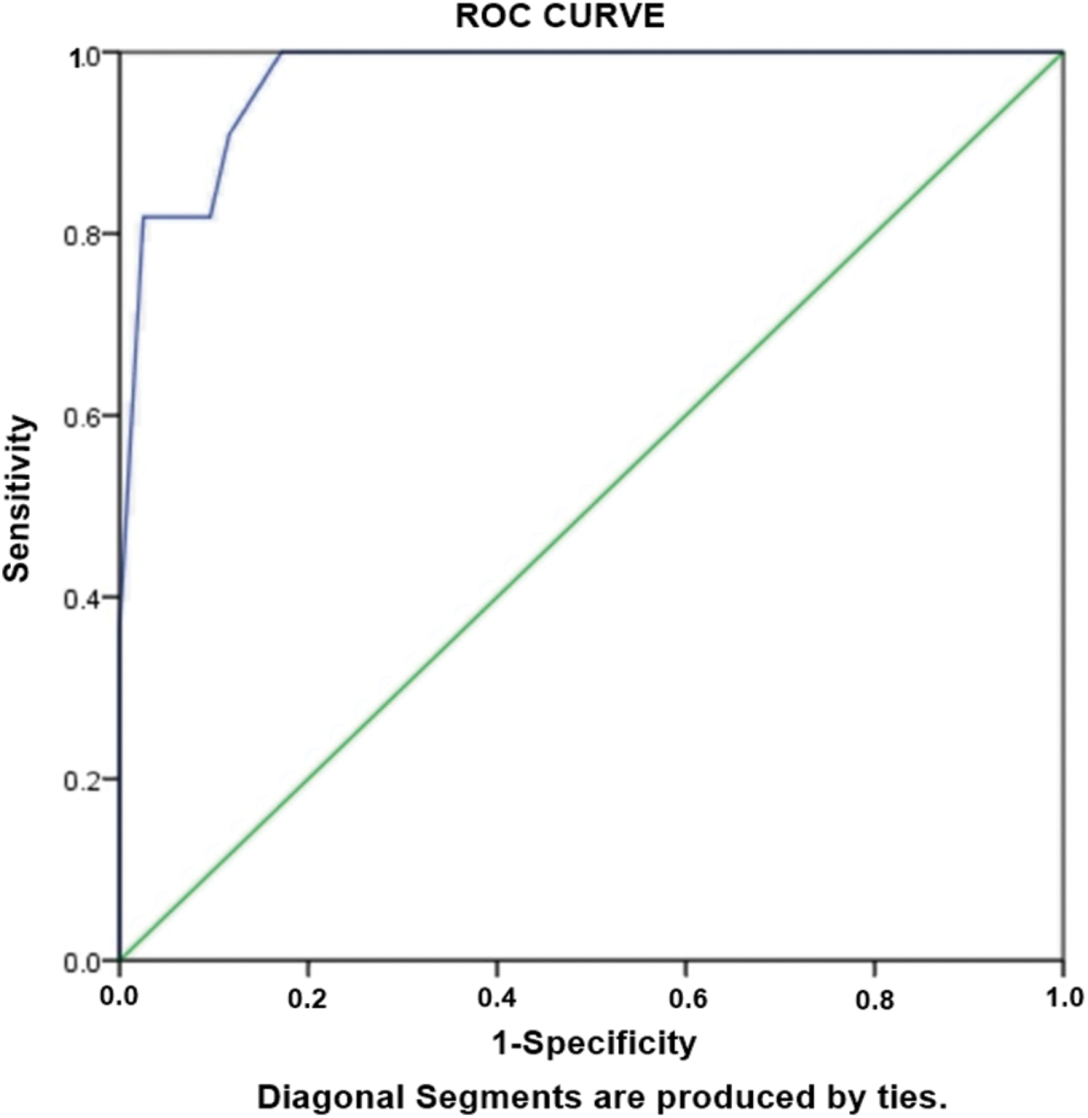
Receiver-operating characteristics curve analysis of multivariate logistic model predicting risk factors of XDR-TB. AUC, area under the curve AUC: 0.972 (95% CI [0.941–1.00], *P* < 0.001).

Aiming to evaluate the risk factors independently associated with development of XDR TB, logistic regression analysis was performed. Statistically significant variables were subjected to unadjusted univariate analysis. The variables with *p* < 0.05 in univariate analysis were considered candidates for adjusted multivariate analysis. We observed that the strongest risk factors independently associated with XDR-TB was cavity on CXR (OR: 59.6), followed by age group of ≤38 years (OR: 13.6), students (OR: 13.0), previous treatment (OR: 12.5) (Table 5). Hosmer-Lameshow test statistics have shown χ2: 4.195, degree of freedom: 5 and *p* value = 0.522, demonstrating model is good fit to data (there is no difference between observed and predicted (model) values of the dependent variable). ROC curve analysis with AUC as 0.972 (95% CI [0.941–1.00], *P* < 0.001) demonstrated excellent predictive ability for regression model of XDR-TB (Fig. 3).

**Table 5:** Univariate and multivariate analysis to evaluate risk factors of XDR-TB.

Variables	Univariate			Multivariate		
	*P*	uOR	95% CI	*P*	aOR	95% CI
≤38 years	0.027	10.4	[1.3–82.9]	0.048	13.6	[1.02–182.1]
Student	0.009	5.2	[1.5–18.5]	0.046	13.0	[1.04–162.0]
Previous treatment	0.001	30.4	[3.7–243.5]	0.029	12.5	[1.3–120.0]
Cavity on CXR	<0.001	63.3	[7.7–514.7]	0.004	59.6	[3.7–966.1]
3+ sputum smear at onset	0.046	4.2	[1.0–17.6]	–	–	–

**Note:**

Hosmer-Lameshow test statistics: χ2: 4.195, degree of freedom: 5, *p* value = 0.522. Only significant associations shown. CXR: chest x-ray.

### Risk factors associated with RR-TB

**Figure 4:**
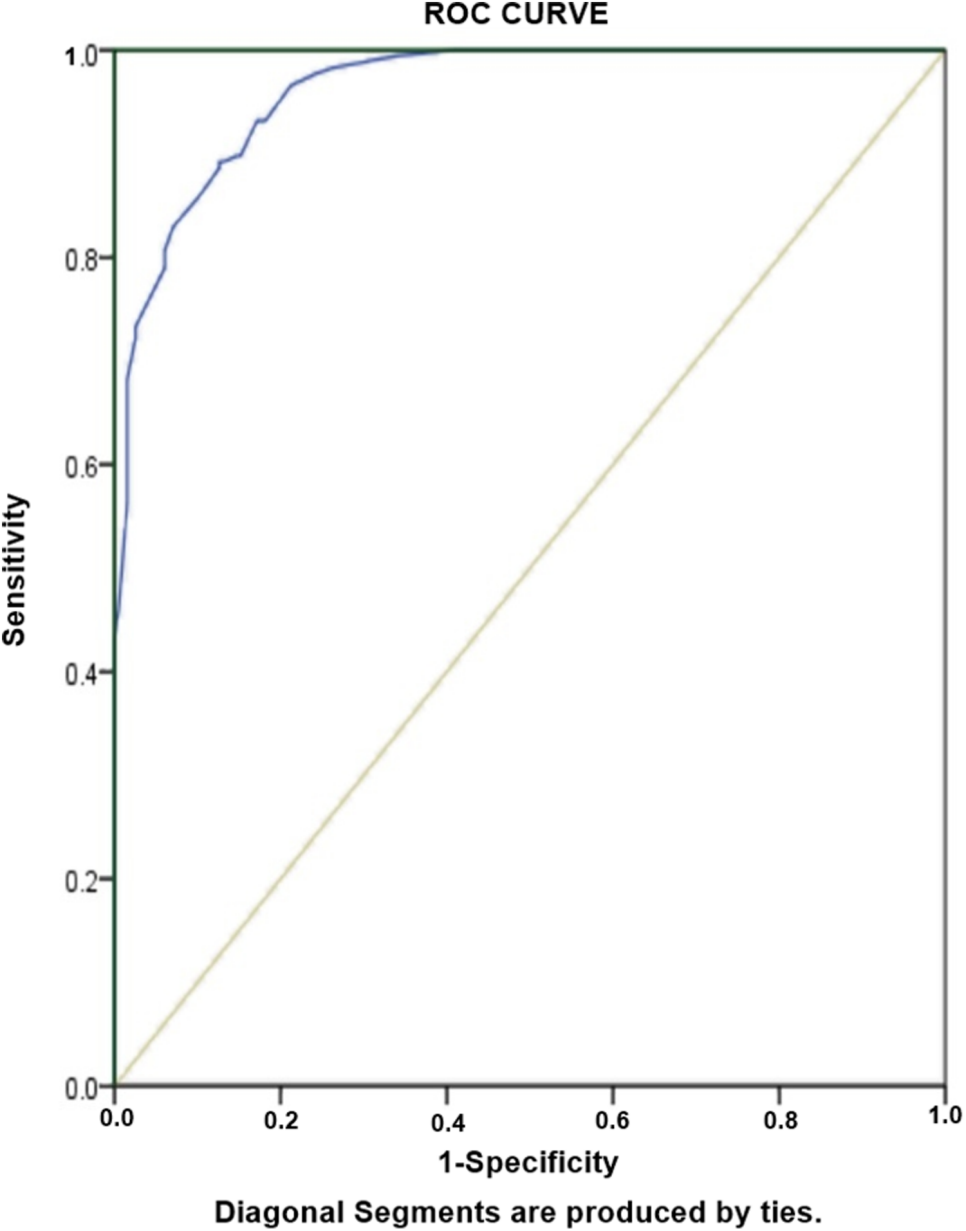
Receiver-operating characteristics curve analysis of multivariate logistic model predicting risk factors of RR-TB. AUC, area under the curve AUC: 0.962 (95% CI [0.946–0.978], *P* < 0.001).

Table 6 Shows results for risk factors associated with RR-TB, amongst diagnosed PTB cases. As would be expected, unemployment (aOR: 41.5) and history of previous TB treatment (aOR: 38.2) are greater risk factors of RR-TB among our study population. Other risk factors of RR-TB included females (OR: 5.7), Failure (OR: 4.9), cavity on chest X-ray (OR: 4.3) and age ≤38 years (OR: 2.8). The predictive accuracy of model was assessed by using Hosmer-Lameshow test statistics. The model is good fit to data (there is no difference between observed and predicted (model) values of the dependent variable) with χ2: 8.4, degree of freedom: 8 and *p* value = 0.39. ROC curve analysis with AUC as 0.962 (95% CI [0.946–0.978], *P* < 0.001) demonstrated excellent predictive ability for regression model of RR-TB (Fig. 4).

**Table 6:** Univariate and multivariate analysis to evaluate risk factors of RR-TB.

Variables	Univariate			Multivariate		
	*P* value	uOR	95% CI	*P*	aOR	95% CI
Age						
≤ 38 years	0.032	1.6	[1.0–2.4]	0.012	2.8	[1.3–6.1]
Gender						
Females	0.034	1.5	[1.0–2.3]	<0.001	5.7	[2.4–13.3]
Area						
Rural	<0.001	2.9	[1.8–4.7]	–		–
Occupation						
Unemployed	<0.001	18.2	[10.8–30.6]	<0.001-	41.5	[16.8–102.9]
Registration group						
Lost to follow-up	0.012	3.4	[1.3–8.8]	–	–	–
Failure	<0.001	13.9	[4.2–46.3]	0.05	4.9	[1.0–25.7]
Family history of TB	0.008	1.9	[1.2–2.9]			
Previous treatment	<0.001	30.4	[16.6–55.8]	<0.001	38.2	[15.1–96.7]
Cavity on CXR	<0.001	3.4	[2.1–5.8]	0.001	4.3	1.9-10.0

**Note:**

Hosmer-Lameshow test statistics: χ2: 8.4, degree of freedom: 8, *p* value = 0.39. Only significant associations shown, CXR: chest x-ray.

## Discussion

To the best of our knowledge, current study is first to accentuate the differential characteristics and risk factors of RR-, MDR- and XDR-TB in Pakistan. It provides important insights into the socio-demographic and clinico-laboratory characteristics of patients with RR-, MDR- and XDR-TB. Following DST analysis, a total 580 patients were stratified into four groups: Controls of DS-TB (*n* = 198), cases of MDR-TB (*n* = 195), cases of XDR-TB (*n* = 11) and RR-TB (*n* = 176). Current study demonstrated the proportion of RR-, MDR- and XDR-TB as 30.3%, 33.6%% and 1.9%, respectively. Previous studies have reported incidence of MDR-TB from 4% to 50% (He et al., 2017; Ullah et al., 2016; Ahmad et al., 2012; Balaji et al., 2010; Liu et al., 2011; Shen et al., 2009; Suarez-Garcia et al., 2009; Lomtadze et al., 2009; Andrews et al., 2011; Barroso et al., 2003; Chuchottaworn et al., 2015), while incidence of XDR-TB was outlined as 37% (Andrews et al., 2011), 23% (Balaji et al., 2010), 3% (He et al., 2017) and 2% (Liu et al., 2011) among PTB patients. These studies have supported our research findings. In present study, patients with age 38 years or less were most likely to have RR-, MDR- and XDR-TB as compared to patients having age greater than 38 years. Age is an important demographic factor contributing to the development of resistance to anti-TB drugs. These findings are consistent with previous studies describing association of MDR- and XDR-TB with younger age groups ranging from 10 to 45 years (Ullah et al., 2016; Ahmad et al., 2012; Balaji et al., 2010; Workicho, Kassahun & Alemseged, 2017; Merza et al., 2011; Mulu et al., 2015). In contrast to our investigations, some studies have not shown any association between DR-TB and age (Andrews et al., 2011; Chuchottaworn et al., 2015; Demile et al., 2018; Ben-Dov & Mason, 1987; Carpenter et al., 1983), or resistance was strongly associated with older age groups of 45–64 years (Suarez-Garcia et al., 2009) and 40–60 years (Zhao et al., 2012). The resistance in young patients might be due to recent transmission, poor adherence to the previous anti-TB medication, due to fear of TB status disclosure (denial of disease) and TB-related stigma. It might also be attributed to forgetfulness due to other activities related to phase of development in which social and cultural assimilation gains prominence in their lives, or financial constraints. This dilemma calls for firm steps to be taken to combat this resistant disease in economically productive population. It is interesting to note that unmarried patients were 11 times more strongly associated with MDR-TB than married patients. Similarly, students were 13 times more strongly associated with XDR-TB in the current study. These findings are in agreement with a previous research (Liu et al., 2011). These findings might be explained by the high prevalence of resistant disease in young age groups or local socioeconomic factors associated to exposure of resistant strains. Several studies have suggested an association between gender and DR-TB. A multicentre case control study conducted in Pakistan, has shown male gender as a risk factor associated with MDR-TB (Ahmad et al., 2012), while some studies have reported female gender as a prognostic factor of MDR TB (Ullah et al., 2016) and XDR-TB (Dalton et al., 2012). Additionally, there are few studies which do not show any association between gender and DR-TB (Demile et al., 2018; Lv et al., 2017). In our study population, females were at 6 times more risk of getting RR-TB. However, our findings have not evidenced any statistically significant relationship between gender, MDR- and XDR-TB. These findings necessitate the further investigations to solve the discrepancies of gender in association with DR-TB. Social behaviour has been proved to be a strong risk factor towards resistant disease (Zhao et al., 2012). A prospective cohort study conducted to determine prevalence and risk factors of resistance to second line drugs revealed smoking and alcohol abuse as strong predictors of the resistance (Dalton et al., 2012). In the present study, regression analysis has shown that tobacco users had three times more odds of MDR-TB than non-users. These findings are consistent with previous report where smoking evolved as a potential predictor of MDR-TB (Fregona et al., 2017). In another investigation from Brazilian state of Ceara, alcohol and smoking had been three times more associated with acquired MDR-TB (Barroso et al., 2003). In a study conducted in India, smoking and alcohol use was associated with low risk of MDR-TB, while the use of alcohol was found to be associated with XDR-TB (Balaji et al., 2010). In current study, there were no alcohol and drug abuse patients in DS-TB controls, while their prevalence among MDR-TB patients was 4.1% and 3.1% respectively and 1.1% for both among RR-TB. This finding might be influenced by bias due to under-reporting of these significant risk factors as a consequence of poor state of general health, lower socio-economic status or early death after developing disease and resistance, prior to the diagnosis. A strong association between history of previous treatment and DR-TB is reported in previous literature (Balaji et al., 2010; Suarez-Garcia et al., 2009; Workicho, Kassahun & Alemseged, 2017; Merza et al., 2011; Zhao et al., 2012; Dalton et al., 2012; Lv et al., 2017; Fregona et al., 2017). Consistent with these investigations, our study has shown a strong association between history of previous anti-TB treatment and DR-TB diseases. These findings infer that routine practice of retreating TB patients without considering DST results is not a constructive approach. It may add to the risk of increasing resistance and ultimate economic burden to patients and healthcare system. Poor outcomes following previous treatment of TB provides a favourable environment for the development of DR-TB. In our investigation, a history of pervious treatment failure was associated with a 5-fold and 9-fold increase in risk of developing RR- and MDR-TB respectively. The possible explanation for this strong association of previous treatment failure in DR-TB groups might be: inappropriate drug regimens; scarce or irregular drug supply; inadequate compliance by patients or clinicians; lack of treatment supervision; poorer access to health-care facilities; and absence of infection control measures in clinics and hospitals. A lack of certainty exists among professionals about the role of lung cavities as a cause or squeal of DR-TB. A cavity can be considered as a cause, because of holding larger count of resistant micro-organisms. Consequently, these resistant mutants undergo fast multiplication due to high oxygenation and protection offered by thick walls against anti-mycobacterial drugs. Moreover, it can also be scrutinized as a consequence of resistant disease because of prolonged duration of active disease catalysing to more lung damage (Barroso et al., 2003). However, the factuality has always been shown the cavities as a cause of DR-TB since the advent of anti-mycobacterial chemotherapy. Our analysis has shown that the presence of cavities on CXR was 4.3 times more associated with RR-TB, 30 times with MDR-TB and 60 times with XDR-TB. This higher association can be justified by the large number of previously treated patients with poor outcomes referred to be a strong predictor of lung cavities. These findings corroborate previous literature showing lung cavities as a notable prognostic factor of resistant disease (Barroso et al., 2003; Mulu et al., 2015). However, a study conducted for risk factors of MDR- and XDR-TB in India demonstrated contradictory findings with no cavitary association of resistant disease upon multivariate analysis (Balaji et al., 2010). Evaluation of risk factors of DR-TB that are obligated for early identification and preferable treatment would represent an immense breakthrough in the care of patients with TB. Effective clinical strategies targeting younger population and strategized protocol of management for retreatment cases can potentially improve the resistance over load, which in turn can translate to reduced economic burden, morbidity and mortality.

### Limitations

Despite first study underscoring the association of various factors with DR-TB in Pakistan, current study is accompanied by few limitations. The retrospective and monocentric study design possesses selection bias. In addition, adults were included in the study population and the results cannot therefore be generalized to paediatric patients. The small number of XDR-TB in the present study may make statistical power quite small for identification of risk factors associated with XDR-TB. However, three previous studies with limited number of DR-TB cases (*N* = 75 (2.1%), 30 (4.3%) and 333 (5%)) evaluating factors of resistance to anti-TB drugs support the findings of the present study (Shen et al., 2009; Suarez-Garcia et al., 2009; Lv et al., 2017). No association was checked between HIV patients and DR-TB due to less number of HIV cases treated in the study setting owing to referral of these critically ill patients to specialized TB care hospitals. Last but not least, current study is strengthened by the first ever investigation on risk factors of XDR-TB in the country and the inclusion of heterogeneous group of patients from NTP. Additionally, the present study improves awareness about risk factors associated with resistance and highlights the need for more future 400 investigations to reduce disease burden.

## Conclusion

Current study demonstrates a sizeable extent of resistant cases among pulmonary TB patients. Expanding pattern of resistance in both previously treated and treatment naïve groups sounded the alarm over the ineffectiveness and inefficiency of NTP. In comparison to DS-TB patients, MDR-TB patients presaged significant risk of resistance among young and unmarried people, smokers, patients with previous treatment history especially with previous treatment failure, and cavitation on chest X-ray. Current analysis also exemplified that XDR-TB occurs more commonly in adults, students with previous treatment history and cavitary disease. Females of young age with unemployment status and history of previous treatment and failure of previous anti-TB therapy, cavitation on chest X-ray are more likely to develop RR-TB. Timely identification of high risk resistant patients will give pronounced advantages regarding proper choices of prevention, treatment and disease management. Attention to these factors, early diagnosis, appropriate treatment regimen, coherent use of DOTS and streamlined infection and transmission control approaches, must be set up to forestall further rise and transmission of resistant disease in comparative settings.

## Supplemental Information

10.7717/peerj.10826/supp-1 Supplemental Information 1 Raw Data Underlying the Results of Study.

10.7717/peerj.10826/supp-2 Supplemental Information 2 Data of Patients with MDR TB used during analysis.

10.7717/peerj.10826/supp-3 Supplemental Information 3 Data of patients with XDR TB used during the analysis.

10.7717/peerj.10826/supp-4 Supplemental Information 4 Code Book.

## Additional Information and Declarations

### Data Availability

The following information was supplied regarding data availability: Raw data are available in the Supplemental Files.
