# Supplementary material for: Evaluation of risk factors associated with the development of MDR- and XDR-TB in a tertiary care hospital: a retrospective cohort study
Source: PeerJ. 2021 Mar 18;9:e10826. doi: 10.7717/peerj.10826 (PMC7982078; doi:10.7717/peerj.10826)
Supplement: Supplemental Information 4 [file peerj-09-10826-s004.pdf]

Code book of MDR

Variable Values

Value		Label
Group	0	Control
	1	Cases
Area	0	Rural
	1	Urban
Tehsil	1	Sialkot
	2	Sambrial
	3	Pasrur
	4	Daska
	5	Other
District	1	Sialkot
	2	Gujranwala
	3	Narowal
	4	Gujrat
	5	Others
AgeDichot	0	38 or less
	1	More than 38
Age1	0	No
	1	Yes
Age2	0	No
	1	Yes
Age3	0	No
	1	Yes
Age5	0	No
	1	Yes
Age4	0	No
	1	Yes
AgeGroups	1	0-14
	2	15-29
	3	30--44
	4	45-59
	5	60 and greater
Gender	0	female
	1	male
Weight	0	less than 38 kg
	1	38 or more
Weight Dichot	0	50 kg or less
	1	more than 50 kg

Marital status	0	Single
	1	Married
	2	Widowed
Single	0	No
	1	Yes
Married	0	No
	1	Yes
Widowed	0	No
	1	Yes
Occupation1	0	Unemployed
	1	Student
	2	Housewife
	3	Businessman
	4	Labour
	7	Others
Unemployed	0	No
	1	Yes
Student	0	No
	1	Yes
Housewife	0	No
	1	Yes
Businessman	0	No
	1	Yes
Labour	0	No
	1	Yes
Others	0	No
	1	Yes
Rgroup1	1	New
	2	Relapse
	3	Lost to follow up
	4	Failure
	5	Others previously treated
New	0	No
	1	Yes
Relapse	0	No
	1	Yes
Lost to followup	0	No
	1	Yes
Failure	0	No
	1	Yes

Others1	0	No
	1	Yes
Previous treatment	0	No
	1	Yes
Other diseases	0	No
	1	Yes
Diabetes	0	No
	1	Yes
Hypertension	0	No
	1	Yes
COPD	0	No
	1	Yes
HCV	0	No
	1	Yes
Tobacco use	0	No
	1	Yes
Alcohol	0	No
	1	Yes
Drugs	0	No
	1	Yes
Family history	0	No
	1	Yes
Radiology	0	No X Ray
	1	Cavitary
	2	Non Cavitary
Diagnostic smear1	0	Negative
	1	Positive
Diagnostic smear	0	Negative
	1	1+
	2	2+
	3	3+
Pos1	0	No
	1	Yes
Pos2	0	No
	1	Yes
Pos3	0	No
	1	Yes
Outcome	1	Cured
	2	Died
	3	Failure

	4	Transfer out
	5	Lost to followup
	6	Not evaluated

Code book of XDR

Variable Values

Value		Label
Group	0	Control
	1	Cases
Area	0	Rural
	1	Urban
Tehsil	1	Sialkot
	2	Sambrial
	3	Pasrur
	4	Daska
	5	Other
District	1	Sialkot
	2	Gujranwala
	3	Narowal
	4	Gujrat
	5	Others
Gender	0	Female
	1	Male
Agedichot	0	40 or less
	1	More than 40
Agegroups	1	0-14
	2	15-29
	3	30-44
	4	45-59
	5	60 and greater
Age1	0	No
	1	Yes
Age2	0	No
	1	Yes
Age3	0	No
	1	Yes
Age4	0	No
	1	Yes

Age5	0	No
	1	Yes
Var00002	0	40 or less
	1	More than 40
Age	0	No
	1	Yes
W8dichot	0	50 kg or less
	1	More than 50kg
Single	0	No
	1	Yes
Widowed	0	No
	1	Yes
Married	0	No
	1	Yes
Marital status	0	Single
	1	Married
	2	Widowed
Education	0	Uneducated
	1	Primary
	2	Middle
	3	Matriculate
	4	Intermediate
	5	Graduate
	6	Masters
	7	Student
Occupation1	0	Unemployed
	1	Student
	2	Housewife
	3	Businessman
	4	Shopkeeper
	5	Labour
	6	Driver
	7	Others
Unemployed	0	Unemployed
	1	Student
	2	Housewife
	3	Businessman
	4	Shopkeeper
	5	Labour
	6	Driver

	7	Teacher	
	8	Others	
	9	Mechanic	
Student	0	Unemployed	
	1	Student	
	2	Housewife	
	3	Businessman	
	4	Shopkeeper	
	5	Labour	
	6	Driver	
	7	Teacher	
	8	Others	
	9	Mechanic	
Housewife	0	Unemployed	
	1	Student	
	2	Housewife	
	3	Businessman	
	4	Shopkeeper	
	5	Labour	
	6	Driver	
	7	Teacher	
	8	Others	
	9	Mechanic	
Businessman	0	Unemployed	
	1	Student	
	2	Housewife	
	3	Businessman	
	4	Shopkeeper	
	5	Labour	
	6	Driver	
	7	Teacher	
	8	Others	
	9	Mechanic	
Labour	0	Unemployed	
	1	Student	
	2	Housewife	
	3	Businessman	
	4	Shopkeeper	
	5	Labour	
	6	Driver	

	7	Teacher
	8	Others
	9	Mechanic
Other	0	Unemployed
	1	Student
	2	Housewife
	3	Businessman
	4	Shopkeeper
	5	Labour
	6	Driver
	7	Teacher
	8	Others
	9	Mechanic
Rgroup1	1	New
	2	Relapse
	3	Lost to follow up
	4	Failure
	5	Others previously treated
New	0	No
	1	Yes
Relapse	0	No
	1	Yes
Lost to followup	0	No
	1	Yes
Failure	0	No
	1	Yes
Others	0	No
	1	Yes
Previous treatment	0	No
	1	Yes
Other diseases	0	No
	1	Yes
Diabetes	0	No
	1	Yes
Hypertension	0	No
	1	Yes
COPD	0	No
	1	Yes
HCV	0	No
	1	Yes

Tobacco use	0	No
	1	Yes
Alcohol	0	No
	1	Yes
Drugs	0	No
	1	Yes
Family history	0	No
	1	Yes
Radiology15	0	No x ray
	1	Cavitary
	2	Non cavitary
Diagnostic smear1	0	Negative
	1	Positive
Diagnostic smear	0	Negative
	1	1+
	2	2+
	3	3+
Outcome	1	Cured
	2	Died
	3	Failure
	4	Transfer out
	5	Lost to followup
	6	Not evaluated
Pos1	0	No
	1	Yes 1+
Pos2	0	No
	1	Yes2+
Pos3	0	No
	1	Yes 3+
